# Clinician Risk Tolerance and Rates of Admission From the Emergency Department

**DOI:** 10.1001/jamanetworkopen.2023.56189

**Published:** 2024-02-16

**Authors:** Peter B. Smulowitz, Ryan C. Burke, Daniel Ostrovsky, Victor Novack, Linda Isbell, Vincent Kan, Bruce E. Landon

**Affiliations:** 1 Department of Emergency Medicine, University of Massachusetts Medical School, Worcester; 2 Milford Regional Medical Center, Milford, Massachusetts; 3 Department of Emergency Medicine, Beth Israel Deaconess Medical Center, Boston, Massachusetts; 4 Soroka University Medical Center, Ben-Gurion University of the Negev, Be’er-Sheva, Israel; 5 Department of Psychological and Brain Sciences, University of Massachusetts, Amherst; 6 Department of Emergency Medicine, University of Massachusetts Medical School, Worcester; 7 Department of Health Care Policy, Harvard Medical School and Division of General Internal Medicine, Beth Israel Deaconess Medical Center, Boston

## Abstract

**Importance:**

Much remains unknown about the extent of and factors that influence clinician-level variation in rates of admission from the emergency department (ED). In particular, emergency clinician risk tolerance is a potentially important attribute, but it is not well defined in terms of its association with the decision to admit.

**Objective:**

To further characterize this variation in rates of admission from the ED and to determine whether clinician risk attitudes are associated with the propensity to admit.

**Design, Setting, and Participants:**

In this observational cohort study, data were analyzed from the Massachusetts All Payer Claims Database to identify all ED visits from October 2015 through December 2017 with any form of commercial insurance or Medicaid. ED visits were then linked to treating clinicians and their risk tolerance scores obtained in a separate statewide survey to examine the association between risk tolerance and the decision to admit. Statistical analysis was performed from 2022 to 2023.

**Main Outcomes and Measures:**

The ratio between observed and projected admission rates was computed, controlling for hospital, and then plotted against the projected admission rates to find the extent of variation. Pearson correlation coefficients were then used to examine the association between the mean projected rate of admission and the difference between actual and projected rates of admission. The consistency of clinician admission practices across a range of the most common conditions resulting in admission were then assessed to understand whether admission decisions were consistent across different conditions. Finally, an assessment was made as to whether the extent of deviation from the expected admission rates at an individual level was associated with clinician risk tolerance.

**Results:**

The study sample included 392 676 ED visits seen by 691 emergency clinicians. Among patients seen for ED visits, 221 077 (56.3%) were female, and 236 783 (60.3%) were 45 years of age or older; 178 890 visits (46.5%) were for patients insured by Medicaid, 96 947 (25.2%) were for those with commercial insurance, 71 171 (18.5%) were Medicare Part B or Medicare Advantage, and the remaining 37 702 (9.8%) were other insurance category. Of the 691 clinicians, 429 (62.6%) were male; mean (SD) age was 46.5 (9.8) years; and 72 (10.4%) were Asian, 13 (1.9%) were Black, 577 (83.5%) were White, and 29 (4.2%) were other race. Admission rates across the clinicians included ranged from 36.3% at the 25th percentile to 48.0% at the 75th percentile (median, 42.1%). Overall, there was substantial variation in admission rates across clinicians; physicians were just as likely to overadmit or underadmit across the range of projected rates of admission (Pearson correlation coefficient, 0.046 [*P* = .23]). There also was weak consistency in admission rates across the most common clinical conditions, with intraclass correlations ranging from 0.09 (95% CI, 0.02-0.17) for genitourinary/syncope to 0.48 (95% CI, 0.42-0.53) for cardiac/syncope. Greater clinician risk tolerance (as measured by the Risk Tolerance Scale) was associated with a statistically significant tendency to admit less than the projected admission rate (coefficient, −0.09 [*P* = .04]). The other scales studied revealed no significant associations.

**Conclusions and Relevance:**

In this cohort study of ED visits from Massachusetts, there was statistically significant variation between ED clinicians in admission rates and little consistency in admission tendencies across different conditions. Admission tendencies were minimally associated with clinician innate risk tolerance as assessed by this study’s measures; further research relying on a broad range of measures of risk tolerance is needed to better understand the role of clinician attitudes toward risk in explaining practice patterns and to identify additional factors that may be associated with variation at the clinician level.

## Key Points

**Question:**

Is clinician risk tolerance associated with the decision to admit from the emergency department?

**Findings:**

In this cohort study utilizing administrative claims data linked to risk tolerance survey results of 691 emergency clinicians in Massachusetts, greater clinician risk tolerance as measured by the Risk Tolerance Scale was associated with a small but statistically significant tendency to admit less than the projected admission rate, whereas the other scales studied had no significant associations.

**Meaning:**

These results suggest that emergency clinician tendencies to admit were minimally associated with risk tolerance as measured by the risk scales used in this study.

## Teaser

This cohort study explores variation in rates of admission from the emergency department and investigates whether clinician risk tolerance is associated with the propensity to admit.

## Introduction

The extent and causes of variation in rates of admission from the emergency department (ED) to the hospital is a topic with important implications for the cost and quality of health care. If rates of admission are too high, in addition to constituting wasteful spending, patients may be exposed to potential harms in the hospital, including risk of iatrogenic harm, nosocomial infection, and deterioration of functional status. In contrast, if rates of admission are too low, patients may be inappropriately discharged without sufficient attention to their presenting condition, potentially resulting in worsening disease, complications, or death. Prior studies have demonstrated substantial variation across regions of the US and across hospitals, even after controlling for the presenting complaint or condition.^1,2,3,4,5,6,7^ There also is meaningful physician-level variation in rates of admission for Medicare patients, even between emergency physicians at the same hospital, suggesting there might be opportunities to devise interventions to support physician decision-making.^8,9^

Closer evaluation of variation at the clinician level is important, as the specific reasons why individual emergency clinicians differ in their practices, particularly after eliminating variation due to differing practice patterns at the hospital level, is poorly understood. Practice patterns with respect to the decision to admit might be influenced by multiple clinician-level factors, including years of experience, comfort with various clinical conditions, and a clinician’s relative tolerance of risk or fear of malpractice. We previously found consistency in rates of admission across clinical conditions among Medicare patients treated by the same physician, suggesting that ED physicians have an overall relatively consistent approach to the decision to admit a patient.^8^ However, that prior analysis may not be generalizable to a non-Medicare population with an overall lower rate of admission. Moreover, the extent to which such variation is mediated by individual clinician risk attitudes is poorly understood.^10^

In this study, we explored 2 complementary aims. First, we sought to extend our previous work examining variation in rates of admission among older adult Medicare patients to further characterize the extent of variation in rates of admission from the ED for a distinct population, and whether admission rates were consistent across key clinical conditions in a non-Medicare population. To accomplish this aim, we used the All Payer Claims Database (APCD) from Massachusetts, which includes data on Massachusetts residents with commercial insurance or Medicaid. Second, we linked the APCD data to data from a statewide survey on risk tolerance that we previously collected from physicians and advanced practice clinicians (APCs) across Massachusetts to evaluate the association between clinician risk tolerance and the propensity to admit in the population included in the APCD.

## Methods

This cohort study was granted institutional review board approval by the Beth Israel Deaconess Medical Center and Harvard Medical School committees on the use of human participants. We followed the Strengthening the Reporting of Observational Studies in Epidemiology (STROBE) reporting guideline.

There were 2 main parts to our analysis. In the first part, we evaluated variation in the rate of admission from the ED, using the difference between the observed and projected admission rates as a measure of deviation from typical clinician practice. We also assessed whether the extent of variation differed across the spectrum of physician admission decisions and the consistency of clinician admission practices across a range of the most common types of medical conditions resulting in admission. This analysis was intended to show whether clinicians have consistent or disparate practices of admission depending on the clinical condition of the patient. In the second part, we evaluated whether the extent to which clinicians deviate from the expected admission rates was associated with their risk attitudes.

### Study Population and Data Source

#### Identifying ED Visits

We used the Massachusetts APCD to identify ED visits from October 2015 through December 2017. The APCD is available from the Center for Healthcare Information and Analysis, an agency of the Commonwealth of Massachusetts and collects information from more than 120 private payers and Medicaid. As a result of the Supreme Court decision in *Gobeille v Liberty Mutual*, as of 2016 self-insured employers are no longer required to submit data and these claims are incompletely represented in the APCD. Despite this ruling, APCD reports that it includes data on coverage and services for the majority of Massachusetts residents under the age of 65 years with public or private insurance.^11^ Though the Centers for Medicare and Medicaid Services prohibit APCDs from sharing data on those covered by Medicare, most patients under the age of 65 years with Medicare also qualify for Medicaid and thus are included in the data set. For this reason, we were able to include some visits in this patient population who are dually eligible but have Medicare as the primary payer.

To identify ED visits, we required a professional claim along with a *Current Procedural Terminology (CPT)* code corresponding to evaluation and management, critical care, or observation care (99281-99285, 99291-99292, 99234-99236, 99217-99220, 99224-99226) and an ED site of service of 23. We excluded patients who were not continuously enrolled in any insurance product for the 1-year period prior to ED visits and for 30 days after for purposes of risk adjustment and for ascertaining outcomes. Following prior work, we grouped ED visit complaints into clinically meaningful categories using the Clinical Classification Software (CCS) for *International Statistical Classification of Diseases and Related Health Problems, Tenth Revision (ICD-10)* available from the Agency for Healthcare Research and Quality (AHRQ). We included all medical conditions for the overall analysis of variation, and for the analysis of consistency of admission practices we focused on the 5 most common types of medical conditions: cardiac, genitourinary, respiratory signs and symptoms, pneumonia, and syncope.

#### Ascertaining Admission Status

For each ED visit, we determined whether the patient was discharged from the ED, admitted to the hospital, or admitted to observation status. Hospitalizations were identified with a facility claim and an inpatient category of claim (11, 18, 21, 28, 41, 65, 66, 84, 86, or 89). Observation stays were identified with a facility claim, not having an inpatient category of claim, and having a *CPT* code of 99218 to 99220, 99234 to 99236, 99224 to 99226, or a revenue code of 0760 or 0762. We considered all transfers and visits under observation status (whether in an ED observation unit or hospital floor) to be equivalent clinically to an admission.

#### Patient Demographic Characteristics

From the member eligibility file and product files, we obtained data on patient demographic characteristics (age ranges: 18-34 years, 35-44 years, 45-54 years, 65-74 years, and 75 years and over; sex; zip code), as well as detailed information on their primary insurance type. We did not have access to race and ethnicity data in the APCD.

#### Risk Tolerance Survey

We augmented the data on ED visits with data on risk tolerance that we collected by survey between January 7, 2020, and September 20, 2020. Our survey was sent to all currently practicing ED attending physicians and ED-based APCs across 54 of 58 acute care hospitals in Massachusetts. The details of the survey collection have been previously described.^10^ Informed consent was obtained at the time of survey completion; clinicians who progressed with the survey beyond the informed consent page were regarded as having provided informed consent. The Beth Israel Deaconess Medical Center granted a waiver of consent for the administrative claims data because it was deidentified data.

The survey instrument included 4 scales: the risk tolerance scale (RTS, 6 items), the stress from uncertainty scale (SUS, 13 items), the fear of malpractice scale (FMS, 6 items), and the need for cognitive closure scale (NCC, 15 items); all scored on a 6-point Likert scale ranging from strongly disagree to strongly agree.^10,12,13,14,15,16,17,18,19^ The survey was administered via the internet with a total of 7 follow-up reminders over 3 weeks. Of 1485 total ED clinicians recruited, 1116 responded (76.5% response rate). Additional information about the survey enrollment and the scales included is detailed in our prior work.^10^ For reference, the full survey is included in the eAppendix in Supplement 1.

#### Linking Procedure

The APCD clinician data file has identifying information for clinicians including the National Provider Identification (NPI), which we used to link to the clinician survey responses. We matched the clinician NPI with the service NPI on the ED visit claim in order to identify the ED clinician who first evaluated each of the visits. If no match was found using service NPI, we then matched to the rendering NPI on that same date. In linking the data on ED visits with the survey data, we excluded clinicians with more than 1 missing survey question and those who saw fewer than 30 patients over the course of the study period. We also excluded ED visits within 30 days of a prior visit. We restricted all analyses to the set of ED visits that were linked to survey respondents. Clinician self-reported race data were collected with categories of Asian, Black, White, and other (which included American Indian or Alaska Native, Pacific Islander, or other).

### Statistical Analysis

To characterize variation in the rates of admission at the clinician level among the population captured in the APCD, we applied a generalized mixed effect linear regression model with a binomial distribution to estimate clinicians’ projected admission rate based on the characteristics and clinical conditions of the patients they treated in the ED. We adjusted for age, sex, Hierarchical Condition Category score, CCS condition, year, month, and day of the week. We also included a random intercept for hospital. This analysis assesses the amount of variation in admission decisions that was not explained by patient diagnosis or other clinical and sociodemographic characteristics. Next, we computed the difference between the observed and projected admission rates as a measure of deviation from the expected rate of admission and plotted it against the projected admission rates to assess the variation. Finally, we used Pearson correlation coefficient to assess whether variation in admission rates was associated with the clinician’s underlying rate of admission (eg, whether or not clinicians with higher rates of admission had more variation than those with lower rates of admission).

To estimate whether clinicians’ admission decisions were consistent across different medical conditions, we used the same aforementioned model to calculate the projected admission rates within the 5 most common medical conditions: nonspecific chest pain; urinary tract infection; respiratory signs and symptoms; pneumonia (except that caused by tuberculosis); and syncope. We then ranked clinicians separately for each condition according to their deviation score and assessed the stability of the ranks by calculating the intraclass correlation coefficient (ICC) across the included conditions. The extent to which the rankings were consistent across the different classes of conditions can be considered a measure of the reliability of physicians’ tendency to admit. A high ICC indicates that there is strong tendency to admit that is present across different conditions, whereas a low ICC indicates that there is variation in the tendency of individual physicians to admit patients with different sets of clinical conditions.

We applied the Spearman correlation test to examine whether the degree physicians deviated from the expected admission rates was associated with the risk tolerance scales. Data were analyzed using SAS version 9.1 (SAS Institute) from 2022 to 2023. We considered 2-sided *P* < .05 to signify statistical significance.

## Results

The total study sample included 392 676 ED visits seen by 691 emergency clinicians. Among the 392 676 patients seen for ED visits, 221 077 (56.3%) were female, and 236 783 (60.3%) were 45 years of age or older; 178 890 visits (46.5%) were for patients insured by Medicaid, 96 947 (25.2%) were for those with commercial insurance, 71 171 (18.5%) were Medicare Part B or Medicare Advantage, and the remaining 37 702 (9.8%) were other insurance category (Table 1). Of the 691 clinicians, 429 (62.6%) were male; mean (SD) age was 46.5 (9.8) years; and 72 (10.4%) were Asian, 13 (1.9%) were Black, 577 (83.5%) were White, and 29 (4.2%) were other race (Table 2).

**Table 1.** Characteristics of Emergency Department Visits

Characteristic	Emergency department visits, No. (%) (N = 392 676)
Admission to hospital	166 887 (42.5)
Age, y	
18-34	60 864 (15.5)
35-54	38 874 (9.9)
45-54	56 545 (14.4)
55-64	68 326 (17.4)
65-74	62 435 (15.9)
≥75	106 022 (27.0)
Sex	
Female	221 077 (56.3)
Male	171 599 (43.7)
Insurance	
Medicare	72 645 (18.5)
Medicaid	182 594 (46.5)
Commercial	98 954 (25.2)
Other	38 482 (9.8)
HCC score, mean	1.6
CCS category	
Symptoms	92 671 (23.6)
Circulatory	98 562 (25.1)
Respiratory	40 446 (10.3)
Digestive	55 975 (14.0)
Genital	42 802 (10.9)
Nervous	16 885 (4.3)
Skin	785 (0.2)
Endocrine	25 916 (6.6)
Infections	785 (2.0)
Blood	785 (2.0)
Neoplasms	353 (0.9)

Abbreviations: CCS, Clinical Classification Software; HCC, Hierarchical Condition Category.

**Table 2.** Clinician Characteristics

Characteristic	No. (%) (N = 691)
Age (quartiles), y	
25-34	91 (13.2)
35-40	162 (23.4)
41-50	220 (31.9)
51-70	217 (31.5)
Sex	
Female	259 (37.4)
Male	432 (62.6)
Years in practice, mean (SD)	15.14 (9.45)
No. of shifts per month, mean (SD)	11.80 (3.80)
Percentage night shifts, mean (SD)	21.29 (29.36)
Clinician payment method	
Salary	152 (22.0)
Salary plus bonus	426 (61.7)
Productivity	83 (12.0)
Other	29 (4.2)
Physician (vs advance practice clinician)	620 (89.7)
Race	
Asian	72 (10.4)
Black	13 (1.9)
White	577 (83.5)
Other^a^	29 (4.2)

^a^ Other race included American Indian or Alaska Native, Pacific Islander.

### Physician Variation

**Figure 1.**
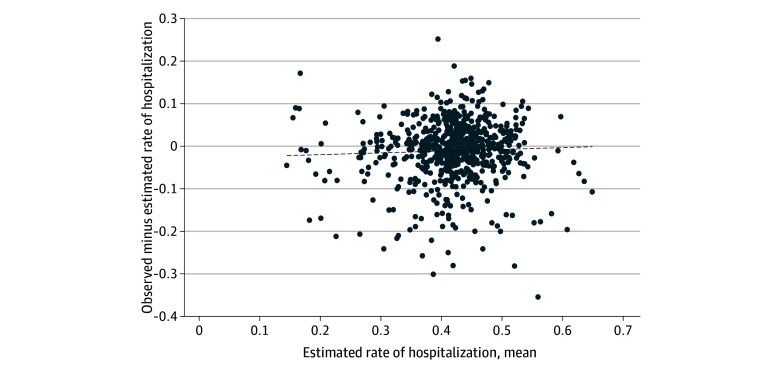
Correlation Between Mean Projected Rate of Hospitalization and Difference Between Actual and Projected Rates

Overall admission rates across the clinicians included ranged from 36.3% at the 25th percentile to 48% at the 75th percentile (median 42.1%). Though we observed substantial variation in admission rates across clinicians, the correlation between the projected rate of admission and the difference between the actual and projected admission rate was statistically nonsignificant (Pearson correlation coefficient = 0.046; *P* = .23). This suggests that across this spectrum of admission likelihood, clinicians were just as likely to over-admit as under-admit compared to their projected rate of admission (Figure 1).

Across specific clinical conditions, the total number of visits and percentage admitted for each category and the range of admission rates across clinicians is shown in Table 3. There was overall weak consistency in admission rates across the most common clinical conditions, with intraclass correlations ranging from 0.09 (95% CI, 0.02-0.17) for genitourinary/syncope to 0.48 (95% CI, 0.42-0.53) for cardiac/syncope (Table 3). The 2 conditions with the highest correlation were cardiac and syncope. These results found that clinicians showed variability in their approach to admission depending on the clinical condition.

**Table 3.** Admission Rates and Intraclass Correlation Analysis of the Top 5 Clinical Conditions

Conditions	No. (%) admitted	Range of admission rates across clinicians, median (IQR)
Cardiac^a^	56 546 (36.7)	0.34 (0.23-0.45)
Genitourinary	28 245 (24.6)	0.24 (0.15-0.33)
Respiratory signs and symptoms	25 915 (23.4)	0.21 (0.11-0.31)
Pneumonia	19 404 (53.3)	0.53 (0.4-0.63)
Syncope	15 898 (41.9)	0.39 (0.25-0.5)
**Conditions**	**ICC (95% CI)^b^**	**ICC^c^**
Cardiac/genitourinary	0.17 (0.10-0.24)	0.16
Cardiac/respiratory signs and symptoms	0.24 (0.16-0.31)	0.24
Cardiac/pneumonia	0.17 (0.09-0.24)	0.17
Cardiac/syncope	0.48 (0.42-0.53)	0.48
Genitourinary/respiratory signs and symptoms	0.30 (0.23-0.37)	0.31
Genitourinary/pneumonia	0.30 (0.24-0.37)	0.30
Genitourinary/syncope	0.09 (0.02-0.17)	0.09
Respiratory signs and symptoms/pneumonia	0.29 (0.22-0.36)	0.28
Respiratory signs and symptoms/syncope	0.14 (0.07-0.21)	0.13
Pneumonia/syncope	0.16 (0.08-0.23)	0.15
Cardiac/genitourinary/respiratory signs and symptoms	0.24 (0.19-0.28)	0.24
Cardiac/genitourinary/pneumonia	0.21 (0.16-0.26)	0.21
Cardiac/genitourinary/syncope	0.25 (0.20-0.30)	0.24
Cardiac/respiratory signs and symptoms/pneumonia	0.23 (0.18-0.28)	0.23
Cardiac/respiratory signs and symptoms/syncope	0.29 (0.25-0.35)	0.28
Cardiac/pneumonia/syncope	0.27 (0.22-0.32)	0.27
Genitourinary/respiratory signs and symptoms/pneumonia	0.30 (0.25-0.35)	0.30
Genitourinary/respiratory signs and symptoms/syncope	0.18 (0.14-0.23)	0.18
Genitourinary/pneumonia/syncope	0.19 (0.14-0.24)	0.18
Respiratory signs and symptoms/pneumonia/syncope	0.20 (0.15-0.25)	0.19

Abbreviation: ICC, intraclass correlation coefficient.

^a^ Cardiac includes (1) nonspecific chest pain and (2) coronary atherosclerosis and other heart disease.

^b^ ICC using rank of ratio, observed and projected.

^c^ ICC using decile of ratio.

#### Admission Rates and Risk Tolerance

**Figure 2.**
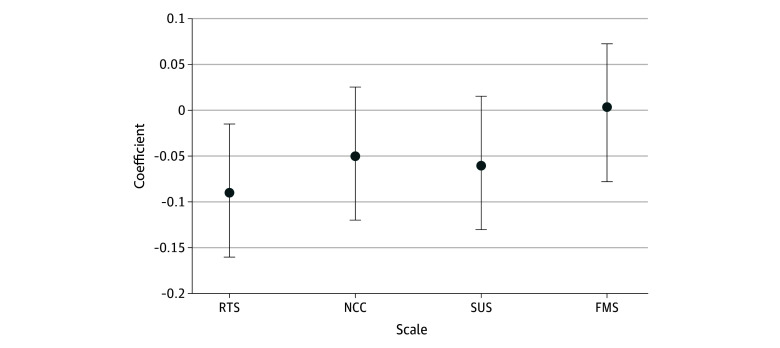
Association of Risk Tolerance Scales With Variance in Actual vs Projected Rate of Admission Error bars denote 95% CIs. FMS indicates fear of malpractice scale; NCC, need for cognitive closure scale; RTS, risk tolerance scale; SUS, stress under uncertainty scale.

Among the 691 clinician respondents, the mean (SD) survey scores were 19.72 (5.06) on the RTS (possible range 6-36 with higher scores indicating greater risk taking), 50.78 (10.37) on the NCC (possible range 15-90 with higher scores indicating greater need for closure), 40.24 (12.10) on the SUS (possible range: 13-78 with higher scores indicating greater stress associated with uncertainty), and 21.67 (6.63) on the FMS (possible range 6-36 with higher scores indicating greater concern about malpractice). In our analysis of the association of risk tolerance as assessed by the survey with variance in the actual vs projected rate of admission, only RTS was found to have any significant association (coefficient, −0.09 [95% CI, −0.16 to −0.02]; *P* = .04). In this case a higher RTS score (greater risk tolerance) was correlated with a tendency to admit less than the projected admission rate. The other scales we studied did not demonstrate any significant association (NCC: coefficient, −0.05 [95% CI, −0.12 to 0.03]; *P* = .15; SUS: coefficient, −0.06 [95% CI, −0.13 to 0.02]; *P* = .10; FMS: coefficient, 0.003 [95% CI, −0.08 to 0.07]; *P* = .92) (Figure 2).

## Discussion

In this statewide analysis of clinician-level variation in admission rates from the ED in Massachusetts, consistent with our prior work we found that there is widespread variation in the propensity to admit, which is not explained by patient case-mix based on observable variables available in claims data. Contrary to our prior work on Medicare patients, however, in this study we found that clinician practices differed across clinical conditions. Finally, by assessing clinician-level rates of admission and surveys on attitudes toward risk tolerance completed by almost 80% of practicing emergency clinicians (physicians and APCs) in Massachusetts, this study’s results found that clinicians with overall greater tolerance to risk—as a general attitude, not specifically related to the provision of medical care—had associated lower rates of admission compared with projected. The other scales that we assessed, however, were not associated with the risk of admission.

The overall variability in admission rates at the level of the individual extends our prior work that demonstrated significant variation in physician-level admission rates for Medicare patients to a younger and more diversely insured population.^8^ Moreover, our results continue to suggest variation both at the hospital and the individual physician level.^9^ However, the finding of lack of stability in the propensity to admit across different types of conditions is contrary to our prior findings in a Medicare population. In general, it seems plausible that clinicians might feel relatively comfortable with some types of conditions as manifested in different rates of admission, but still demonstrate a tendency compared with their peers toward higher or lower admissions rates based on their innate risk preferences (as suggested by the RTS findings). It is also possible that these results are being affected by the overall lower rate of admissions in this largely Medicaid and commercially insured and younger population compared with a Medicare population, and thus deserves further study.

When exploring reasons for variation at the individual level, we focused on attitudes toward risk as measured by our statewide survey of Massachusetts ED clinicians. In contrast to our expectations, we found that only 1 of our 4 measures of risk tolerance was significantly associated with a clinician’s propensity to admit. However, even this association is of interest, especially given that the RTS is associated with risk preferences outside the scope of medical care. As such, it is possible that the responses to the RTS survey may be better capturing clinicians’ overall risk preferences, especially if clinicians are biased to over- or underestimate their level of clinically related risk for reasons related to social desirability bias. There are several additional possible explanations for this finding. First, it could suggest that other clinician-level factors are more heavily affecting the final determination of the decision to admit such that risk tolerance is not a key factor in this decision. It is also possible that risk-averse clinicians are ordering significantly more tests (eg, laboratory tests, imaging, stress tests) in evaluating patients presenting for emergency care, which mitigates the risk associated with discharge. Third, as previously noted, it is possible that the survey did not fully capture clinician risk attitudes and tolerance. Though the 4 scales we used have been well validated, it is possible that social desirability bias is affecting the survey results.^20^ That is, especially risk-averse (or tolerant) clinicians may have responded to survey questions in a way that would seem more acceptable to their peers as opposed to reporting their true risk preferences. Clinician self-assessment of their own skillset or personality may also be inaccurate.^21^ Violato et al^22^ found that physicians in the lowest quartile overestimated and in the highest quartile underestimated their skills in patient management, clinical assessment, professional development, and communication. Dolar et al^23^ reported that peer assessment of physician personality had greater predictive validity than self-assessment. Furthermore, Andreatta et al^24^ found that self-assessment of surgical skillset was unreliable compared with objective measurement of skills. These studies suggest that other objective measures combined with peer assessment of risk tolerance may be more reliable, though the extent to which they project actual practice patterns is key.

### Limitations

There are key limitations to our study. First, the practice data we used from the APCD predates our survey data by 3 to 5 years. We note that this gap existed in several other studies using these scales, with time lags ranging from 6 months to 5 years.^25,26,27,28,29^ Furthermore, evidence demonstrates that the RTS scale has considerable stability with test-retest stability at 13 weeks of 0.79.^15^ However, we are unable to fully rule out the possibility that clinicians’ responses to the scales could change over time.

Next, because self-insured plans are not required to provide data to the APCD, the population of patients was heavily Medicaid. This may have affected our findings and may not be generalizable to a larger population of commercially insured or Medicare patients. The data sharing limitations also led us to utilize a different statistical method for evaluating the consistency in variation of admission rates across medical conditions. In this study we used ranks obtained from aggregated data to calculate the ICC instead of reporting the correlation between random effects in a mixed-effect model with physicians and medical conditions as random terms (adjusted for potential confounders). It is possible that these methods resulted in differing results for the degree of variation or the consistency across conditions, although each accounted for correlations at the level of the clinician as well as random statistical noise. Also, with respect to the ICC scores, it is possible that the differences between this younger (the mean patient age from our prior work was 72.5 years) and mainly commercially insured and Medicaid or dually eligible population is not generalizable to an exclusively Medicare population, hence the distinction from our prior work. Similarly, we may find a different result when evaluating the association between the risk tolerance scales and admission rates in a Medicare population. As is the case with all studies using claims data, our ability to control for severity is limited by the lack of information on vital signs, triage classification, and other clinical variables. Clinicians may be self-selecting to a higher or lower severity of patient population by where they choose to work, what shifts they work, or what patients they choose to see, though including fixed effects for hospitals should mitigate this concern related to clinician choice of setting. Additionally, though we used validated scales to assess clinician risk tolerance, the surveys are subject to nonresponse as well as socially desirable responses biases.

## Conclusions

In summary, in this retrospective cohort study of clinician risk tolerance and admission rates from the ED in Massachusetts, we found meaningful variation among ED clinicians in admission rates. In contrast to what we observed in an older Medicare population, however, there was less consistency across different conditions. Moreover, these tendencies were associated with 1 of 4 of our innate risk tolerance measures. Furthermore, research relying on a broad range of measures of risk tolerance is needed to better understand the specific role of clinician attitudes toward risk in explaining practice patterns, (eg, the ordering of certain tests or performing key procedures in the ED), and to identify additional factors that may be driving variation at the clinician level.

## Notes

Supplement 1. **eAppendix.** Survey Instrument

Supplement 2.
Data Sharing Statement
