# Supplementary material for: Clinician Risk Tolerance and Rates of Admission From the Emergency Department
Source: JAMA Netw Open. 2024 Feb 16;7(2):e2356189. doi: 10.1001/jamanetworkopen.2023.56189 (PMC10873771; doi:10.1001/jamanetworkopen.2023.56189)
Supplement: Supplement 1. — eAppendix. Survey Instrument [file jamanetwopen-e2356189-s001.pdf]

Supplemental Online Content

Smulowitz PB, Burke RC, Ostrovsky D, et al. Clinician risk tolerance and rates of admission from the emergency department. *JAMA Netw Open.* 2024;7(2):e2356189. doi:10.1001/jamanetworkopen.2023.56189

eAppendix. Survey Instrument

This supplemental material has been provided by the authors to give readers additional information about their work.

© 2024 Smulowitz PB et al. *JAMA Network Open.*

eAppendix: Survey instrument

Please indicate the extent to which you agree or disagree with each of the following statements (1 – Strongly Disagree; 2- Moderately Disagree; 3 – Slightly Disagree; 4 – Slightly Agree; 5 – Moderately Agree ; 6 – Strongly Agree)

Risk-Taking Scale

1. I enjoy taking risks.
2. I try to avoid situations that have uncertain outcomes.
3. Taking risks does not bother me if the gains involved are high.
4. I consider security an important element in every aspect of my life.
5. People have told me that I seem to enjoy taking chances.
6. I rarely, if ever, take risks when there is another alternative.

Need for Cognitive Closure Scale

7. I don't like situations that are uncertain.
8. I dislike questions which could be answered in many different ways.
9. I find that a well-ordered life with regular hours suits my temperament.
10. I feel uncomfortable when I don't understand the reason why an event occurred in my life.
11. I feel irritated when one person disagrees with what everyone else in a group believes.
12. I don’t like to go into a situation without knowing what I can expect from it.
13. When I have made a decision, I feel relieved.
14. When I am confronted with a problem, I’m dying to reach a solution very quickly.
15. I would quickly become impatient and irritated if I would not find a solution to a problem immediately.
16. I don’t like to be with people who are capable of unexpected actions.
17. I dislike it when a person’s statement could mean many different things.
18. I find that establishing a consistent routine enables me to enjoy life more.
19. I enjoy having a clear and structured mode of life.
20. I do not usually consult many different opinions before forming my own view.
21. I dislike unpredictable situations.

22. (Additional item) In my day to day practice, I am fearful of making a mistake which results in harm to the patient.

Stress from Uncertainty Scale

23. The uncertainty of patient care often troubles me.
24. Not being sure of what is best for a patient is one of the most stressful parts of being a health care provider.
25. I am tolerant of the uncertainties present in patient care.
26. I find the uncertainty involved in patient care disconcerting.
27. I usually feel anxious when I am not sure of a diagnosis.

© 2024 Smulowitz PB et al. *JAMA Network Open.*

28. When I am uncertain of a diagnosis, I imagine all sorts of bad scenarios – patient dies, patient sues, etc.
29. I am frustrated when I do not know a patient's diagnosis.
30. I fear being held accountable for the limits of my knowledge.
31. Uncertainty in patient care makes me uneasy.
32. I worry about malpractice when I do not know a patient's diagnosis.
33. The vastness of the information that physicians are expected to know overwhelms me.
34. I frequently wish I had gone into a specialty or subspecialty that would minimize the uncertainties of patient care.
35. I am quite comfortable with the uncertainty in patient care

36. (Additional item) In my day to day practice, I am fearful of making a mistake which results in being sued.

Fear of Malpractice Scale

37. I have had to make significant changes in my practice pattern because of recent legal developments concerning medical delivery.
38. I am concerned that I will be involved in a malpractice case sometime in the next 10 years.
39. I feel pressured in my day-to-day practice by the threat of malpractice litigation.
40. I order some tests or consultations simply to avoid the appearance of malpractice.
41. Sometimes I ask for consultant opinions primarily to reduce my risk of being sued.
42. Relying on clinical judgment rather than on technology to make a diagnosis is becoming riskier from a medicolegal perspective.

Lastly, please complete the brief demographic and work-related questions below.
43. What is your age?
44. Are you Hispanic, Latino/a, or Spanish origin? Y/N
45. Which one or more of the following would you say is your race? [ ] White   [ ] African-American   [ ] Asian   [ ] American-Indian/Alaska Native   [ ] Pacific Islander   [ ] Other – please specify:
46. What is your gender? Male/Female
47. Are you an: MD, DO, NP, PA
48. Number of years of practice after graduating residency (or APP training)
49. On average, how many shifts per month do you work?
50. On average, what percent of your total shifts are night shifts?
51. What is the method of reimbursement for you at your primary practice site: a) Salary; b) Salary plus incentive bonus; c) Pure productivity basis; d) other (please define)

© 2024 Smulowitz PB et al. *JAMA Network Open.*
