# Supplementary material for: Clinician Risk Tolerance and Rates of Admission From the Emergency Department
Source: JAMA Netw Open. 2024 Feb 16;7(2):e2356189. doi: 10.1001/jamanetworkopen.2023.56189 (PMC10873771; doi:10.1001/jamanetworkopen.2023.56189)
Supplement: Supplement 2. — Data Sharing Statement [file jamanetwopen-e2356189-s002.pdf]

Data Sharing Statement

Smulowitz. Clinician Risk Tolerance and Rates of Admission From the Emergency Department. *JAMA Netw Open*. Published February 15, 2024. doi:10.1001/jamanetworkopen.2023.56189

Data
Data available: No

Additional Information
Explanation for why data not available: data comes from statewide database and sharing is not permitted by regulation. aggregated data can be available upon request.
